# ﻿Bivalves of superfamily Galeommatoidea (Mollusca, Bivalvia) from western South Africa, with observations on commensal relationships and habitats

**DOI:** 10.3897/zookeys.1207.124517

**Published:** 2024-07-22

**Authors:** Paul Valentich-Scott, Charles Griffiths, Jannes Landschoff, Ruiqi Li, Jingchun Li

**Affiliations:** 1 Santa Barbara Museum of Natural History, 2259 Puesta del Sol Road, Santa Barbara, CA 93105, USA; 2 Department of Biological Sciences, University of Cape Town, Private Bag X3, Rondebosch 7701, South Africa; 3 Sea Change Trust, Cape Town, Western Cape, South Africa; 4 Department of Botany and Zoology, Stellenbosch University, Private Bag X1, Matieland, 7602, South Africa; 5 Department of Ecology and Evolutionary Biology, University of Colorado Boulder, 1900 Pleasant Street, Boulder, CO 80309, USA; 6 Museum of Natural History, University of Colorado Boulder, 265 UCB, Boulder, CO 80309, USA

**Keywords:** Biodiversity, commensalism, heart urchin, South Atlantic Ocean, *Spatagobrissusmirabilis*, *Spatanguscapensis*, symbiosis, taxonomy

## Abstract

The Galeommatoidea are a diverse but little-studied group of small bivalves, well known for the symbiotic relationships many species have with a range of invertebrate taxa. Four species collected from the Western Cape region of South Africa were examined and illustrated, providing new details on their habitat preferences, and depicting the mantle structure of live specimens for the first time. *Brachiomyaducentiunus***sp. nov.**, is described herein, and an additional record of *Montacutasubstriata* (Montagu, 1808) is reported from South Africa. *Brachiomyaducentiunus* and *Montacutasubstriata* have obligate symbiotic relationships with different burrowing echinoids, while *Kelliabecki* (WH Turton, 1932) and *Melliteryxmactroides* (Hanley, 1857) are free-living. DNA data and phylogenetic analyses are provided for three of the species.

## ﻿Introduction

This study examines four galeommatoidean species collected near Cape Town, South Africa, mostly from the intertidal or inshore zones, but one from a depth of 122 m. We report on the characteristics and habits of each of these species, one of which is described as new to science.

The Galeommatoidea are perhaps the most poorly known, yet most diverse, of all groups of marine bivalve mollusks (Morton and Valentich-Scott 1989; Bouchet et al. 2002; Goto et al. 2012; Li et al. 2012; Huber 2015; Li et al. 2016), largely due to their small sizes, cryptic lifestyles, and frequent specialized symbiotic relationships with a wide diversity of marine invertebrate taxa.

The unusual lifestyles of Galeommatoidea have piqued the interest of scientists for more than two centuries. Turton (1822) noted that *Montacutasubstriata* (Montagu, 1803) was found abundantly on an echinoid, attaching to its spines using “slender filaments issuing from the middle margin” (i.e., the byssal threads). Récluz (1844) further added to the knowledge of commensal relationships of various “*Erycina*” species (Galeommatoidea) and their invertebrate hosts. During the twentieth century a vast number of publications including pivotal papers by Yonge (1952), Morton (1962), Oldfield (1964), Gage (1966), and Morton and Valentich-Scott (1989) described the commensal relationships and functional morphology of various galeommatids.

The current century has seen a continuing high level of interest in galeommatoid biology, with many more details published about their host relationships and interactions (Lützen and Nielsen 2005); Passos and Domaneschi 2006; Rotvit et al. 2007). In the past 15 years considerable work has also been published on the phylogeny of the Galeommatoidea, along with a large expansion in our knowledge of the diversity of invertebrates with which they interact (Goto et al. 2012; Li et al. 2012). Huber (2015) also documented the Galeommatoidea globally, based on conchological characteristics. These studies revealed the morphological disparity and convergence of different galeommatoidean groups and presented new challenges to taxonomical classifications in this bivalve superfamily. These works also highlighted geographical gaps in our understanding of galeommatoidean diversity.

Zettler and Hoffman (2021) described and illustrated six galeommatoidean species from shallow to deep water off Namibia, Africa. Of the six species, four were new to science, indicating much is still unknown in the fauna of southwest Africa. Surprisingly, one of the recorded galeommatids, *Kurtiellabidentata* (Montagu, 1803), is also a fairly common, free living associate within ophiuroid-dominated communities in the North Atlantic (Oliver et al. 2016).

A number of authors have included descriptions or coverage of galeommatoidean bivalves in the course of broader treatments of the South African mollusk fauna. Sowerby’s ‘Marine Shells of South Africa’ (1892) included six species of galeommatids within the genera *Lasaea* Brown, 1827; *Kellia* Turton, 1822; and *Montacuta* Turton, 1822. Smith (1904) provided an extensive checklist of mollusks in the Port Alfred region, on the South African south coast, which included dozens of new species descriptions, including one species each of *Lepton* and *Tellimya*. In the most extensive account of South African galeommatids to date, Bartsch (1915) documented 25 species, of which 15 were new to science. This account was followed by Turton (1932), who documented an impressive 54 galeommatids, including 16 new species, mostly also from the Port Alfred region. Barnard (1964a) gave a detailed account of 14 galeommatids in his overview of South African mollusks and subsequently added two new genera and two new species from the region (Barnard 1964b). As their book on Southern African shells (which is otherwise the most comprehensive account available on the regional mollusk fauna) excludes many of the smaller bivalves, Kilburn and Rippey (1982) report on only five galeommatoideans, despite the fact that their publication covered the wider Southern African region (including Namibia and southern Mozambique).

## ﻿Materials, methods, and abbreviations

### ﻿Study locality

The area for the present study of galeommatoidean species has a mild Mediterranean subtropical climate, with warm, dry summers and cooler wet winters. The oceanographic regime of the wider region, the recognized biogeographic regions in the area, and the resulting patterns of marine biodiversity and endemicity are all described by Griffiths et al. (2010). A more detailed account of the physical oceanography of False Bay, one of few significant embayments along the almost linear South African coastline and the collection site of three of the four species covered in this report, is described in detail by Gründlingh and Largier (1991). A description of the coastal fauna of the broader Atlantic coastline of Southern Africa is provided by Branch and Griffiths (1989). The habitat of this region is vastly influenced by extensive kelp forests that grow along rocky reefs and intermixed sandy beaches and are altogether informally known as the “Great African Seaforest.”

### ﻿Abbreviations

The following abbreviations represent institutions at which we examined type specimens, reviewed images, or deposited voucher specimens of this study’s subject species.

**NHMUK** The Natural History Museum, London, UK

**NMSA** KwaZulu-Natal Museum, Kwazulu-Natal, South Africa

**SAM** Iziko South African Museum, Cape Town, South Africa

**SBMNH**Santa Barbara Museum of Natural History, Santa Barbara, California, USA

**UCM**Museum of Natural History, University of Colorado, Boulder, Colorado, USA

**USNM**United States National Museum collection in the National Museum of Natural History, Smithsonian Institution, Washington, D.C., USA

**ZC** Oxford Museum of Natural History, Oxford, UK

### ﻿Examined taxa

The samples of *Brachiomyaducentiunus*, new species, were collected in 2016 and 2018 from Miller’s Point, in False Bay on the Cape Peninsula (34.13°S, 18.28°E) by freediving and hand excavating specimens of its host, the burrowing echinoid *Spatagobrissusmirabilis* H. L. Clark, 1923, from coarse sand sediments in a water depth of approximately 3 m. Each echinoid was placed immediately after collection into a plastic bag and brought alive to an adjacent field laboratory, where symbiotic species were removed, counted, and preserved. The samples, including the type specimens, were collected by Jannes Landschoff, Craig Foster, and Charles Griffiths.

Samples of *Montacutasubstriata* were found crawling on the oral surface spines of the heart urchin *Spatanguscapensis* Döderlein, 1905, collected from a trawl sample on the RS *Africana* (cruise AFR289) during the 10 October 2016 austral spring demersal research survey (Trawl 094, Station No A32843, 122 m) at Agulhas Bank, approximately 110 km south off Mossel Bay (35.196°S, 22.056°E). The cruise was jointly organized by the former South African Departments of Agriculture, Forestry and Fisheries (DAFF) and the Department of Environmental Affairs (DEA), now merged into the Department of Forestry, Fisheries and the Environmental (DFFE). Samples were obtained from a single urchin by Jannes Landschoff using a German otter trawl design and a 75 mm mesh cod-end fitted with a 35 mm mesh liner (see Atkinson et al. 2011).

*Kelliabecki* were collected by hand from beneath boulders in a mid-intertidal rock pool at Glencairn, on the east coast of the Cape Peninsula (34.162°S, 18.432°E), collected by Charles Griffiths in 2020.

*Melliteryxmactroides* were collected at Miller’s Point in the small intertidal section north of the tidal pool (34.231°S, 18.476°E), collected by Jannes Landschoff in 2020.

Before being preserved in 96% ethyl alcohol, all samples were photographed alive, either in situ prior to collection or in the laboratory while still alive. We used either an Olympus Tough digital camera on microscope setting or a digital SLR camera and macro lens.

### ﻿DNA amplification

Genomic DNA of the four species were extracted from mantle, foot or whole body of the specimens using the E.Z.N.A. Mollusc DNA Kit (Omega Bio-tek) following manufacturer’s instructions. DNA concentrations were assessed using the Qubit Fluorometer (Invitrogen). Fragments of two nuclear genes (28S rRNA, Histone H3) and one mitochondrial gene (16S rRNA) were amplified for phylogenetic analyses. The 28S gene fragment was amplified using primers D23FLas (5’-CCGCATAGAGGCAAACGGGT-3’) (Li et al. 2013) / D6R (5’-CGAAGTTTCCCTCAGGATAGCTGG-3’) (Park and Ó Foighil 2000), following a standard PCR protocol with an annealing temperature at 52 °C. The 16S gene fragment was amplified using primers 16SLasF (5’-TAGATTAAGGGTTGGGCCTG-3’)/16SLasR (5’-GCCTAAATGGTAAGACTGTTCG-3’) (Valentich-Scott et al. 2013) following a touchdown protocol. The initial annealing temperature was 55 °C, and was decreased by 2 °C per cycle, until the final annealing temperature 48 °C was reached. The H3 gene was amplified using primers H3F (5’-ATGGCTCGTACsdCAAGCAGACVGC-3’)/H3R (5’-ATATCCTTRGGCATRATRGTGAC-3’) (Li et al. 2016) with an annealing temperature at 52 °C. PCR products were sequenced by Quintara Biosciences and DNA sequences were deposited in GenBank (See Table 1 for accession numbers).

**Table 1.** GenBank ID of successfully amplified DNA fragments from the four galeommatoidean species. Note that some species included multiple individuals from the same voucher lot. A dash indicates failed PCR amplification.

Voucher SBMNH	Species	28S	16S	H3
665157	*Montacutasubstriata*	–	–	–
665157		–	–	–
666951	*Kelliabecki*	–	PP431564	–
666970	*Brachiomyaducentiunus*	–	PP431565	PP454116
666970		–	PP431566	PP454117
666970		–	PP431567	PP454118
665156	*Melliteryxmactroides*	PP431562	PP431568	PP454119
665156		PP431563	PP431569	PP454120

### ﻿Phylogenetic analyses

Due to specimen sizes and preservation condition, not all specimens amplified for all three genetic markers. Table 1 summarizes the PCR amplification results.

The same three genetic markers from other galeommatoidean species belonging to closely related genera were downloaded from GenBank (Suppl. material 1) that included many unidentified galeommatid taxa from the non-systematic paper by Li et al. (2016). Phylogenetic positions of *Kelliabecki*, *Brachiomyaducentiunus*, and *Melliteryxmactroides* in relationship with other species were assessed. For each genetic marker, sequences were aligned using MUSCLE 5.1 (edgar) implement in Geneious Prime 2023.2.1. Alignments of multiple genetic markers were concatenated when applicable. Maximum likelihood inferences were performed using RAxML 8.2.11 (Stamatakis et al. 2008) with the GTR CAT model and 100 bootstrap replicates. Bayesian phylogenies were constructed using MrBayes 3.2.6 (Huelsenbeck and Ronquist 2001) using the GTR model. For each dataset, two independent runs were performed for 100,000 generations with a 10,000 burn-in. Cumulative split frequencies were observed to be below 0.01 to ensure convergence.

## ﻿Results

### ﻿Systematic Account

**Superfamily Galeommatoidea Gray, 1840**

**Family Lasaeidae Gray, 1842**

#### ﻿ Brachiomya

Taxon classificationAnimaliaGaleommatidaMontacutidae

Jespersen et al., 2004

D8F382D2-3D45-50A2-B527-03FF201C7D1C

Brachiomya Jespersen, Lützen & C. Nielsen, 2004. Type species (original designation) Solecardiastigmatica (Pilsbry, 1921). Recent.

##### Description.

Shell small (less than 5 mm); transversely ovate; anterior end narrower than posterior end; inequilateral; hinge plate narrow; both valves with a small triangular pseudocardinal tooth beneath umbos; ligament internal, in oblique resilifer; mantle folds wrapped over most of the shell and anteriorly extended into a large inhalant-pedal siphon, posteriorly into a smaller exhalent siphon; mantle with many slender, terminally spatulate tentacles; foot elongate; gills with inner demibranch only.

##### Discussion.

Jespersen et al. (2004) distinguished *Brachiomya* from other galeommatoidean genera by the presence of extensive mid-mantle folds with many spatulate tentacles. Huber (2015) accepted *Brachiomya* as a monotypic genus. As Jespersen and Huber both postulated, we have found *Brachiomya* to group with members of the Lasaeidae. All species are likely to be obligate commensals with echinoid echinoderms.

#### ﻿ Brachiomya ducentiunus sp. nov.

Taxon classificationAnimaliaGaleommatidaMontacutidae

887FD09C-0429-5E11-A580-FB578A3EFEB5

https://zoobank.org/25211096-71D8-41CA-A5CF-DADEDA4CD18B

**Figure 1.**
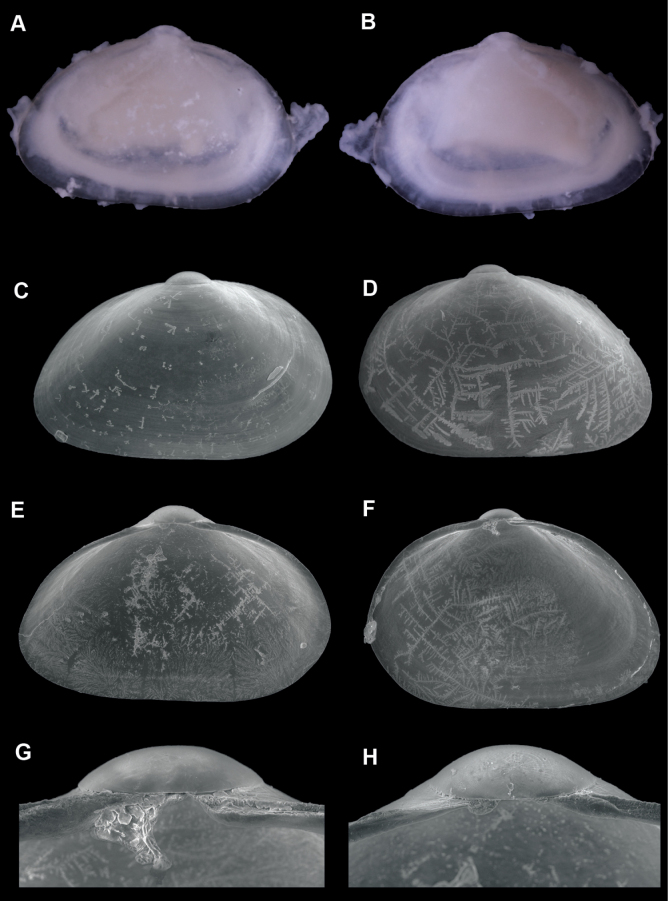
*Brachiomyaducentiunus* sp. nov. **A, B** holotype, SBMNH 713162, length 2.50 mm, height 1.75 mm **A** exterior of right valve **B** exterior of left valve **C–H** paratypes, SBMNH 666970, crystallization on exterior and interior of shell is from dried bleach, not a sculpture element **C** exterior of right valve, length 2.17 mm, height 1.38 mm **D** exterior of left valve, length 2.40 mm, height 1.56 mm **E** interior of left valve, length 1.87 mm, height 1.19 mm **F** interior of right valve, length 2.42 mm, height 1.67 mm **G** hinge of right valve **H** hinge of left valve.

**Figure 2.**
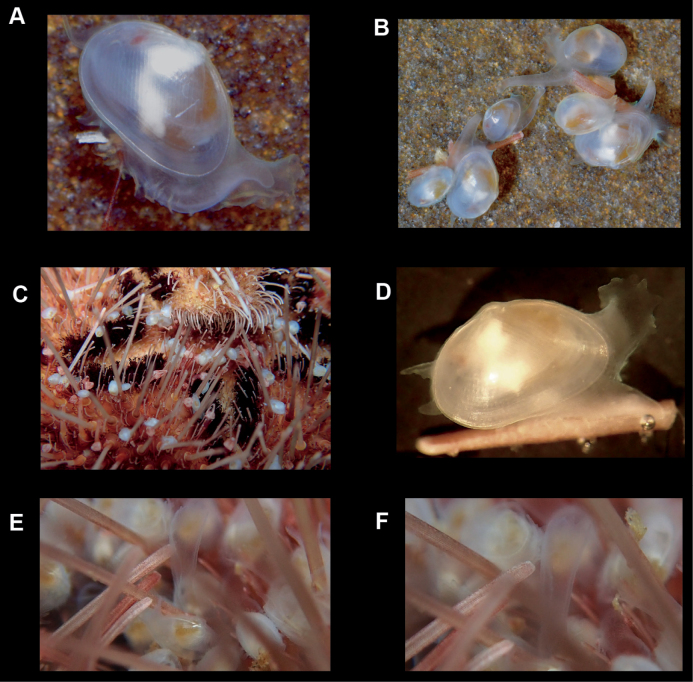
*Brachiomyaducentiunus* new species, living animals **A, B** crawling on hard substrate **C** overview of specimens crawling on the aboral surface of urchin *Spatagobrissusmirabilis***D** crawling on urchin spine with mantle and foot extended **E, F** extended mantle between urchin spines.

Figs 1A–H , 2A–F , Suppl. material 2

##### Type locality.

Miller’s Point Lagoon, in False Bay, Western Cape Province, South Africa; 34.231°S, 18.477°E; 3 m; attached to spines, or crawling amongst spines of *Spatagobrissusmirabilis* (Clark, 1923), collected by Charles Griffiths, July 2018.

##### Type material.

***Holotype*** (Fig. 1A, B), SBMNH 713162, length 2.50 mm, height 1.75 mm, preserved in 70% EtOH. 13 ***Paratypes***, SAMC-A096817, same locality as holotype, largest specimen length 2.5 mm, height 1.7 mm, preserved in 70% EtOH, collected by Jannes Landschoff and Craig Foster, 9 June 2016. 7 ***Paratypes***, SBMNH 666970, same locality and collector as holotype (Fig. 1C–H), dried specimens mounted on SEM stub; length 2.17 mm, height 1.38 mm; length 2.40 mm, height 1.56 mm; length 1.87 mm, height 1.19 mm; length 2.42 mm, height 1.67 mm. 1 ***Paratype***, UCM 60476; length 1.5 mm, height 1.0 mm.

##### Description.

Shell extremely thin, fragile, moderately inflated, translucent; inequilateral, slightly longer anteriorly; ovate-elongate; anterior end obliquely truncate in larger specimens; posterior end broadly rounded; ventral margin straight, slightly invaginated in some; dorsal margin gently sloping from umbos; shell margins weakly gaping; prodissoconch well defined, umbonate, smooth, subcircular; prodissoconch length ~ 350 μm; external sculpture of commarginal striae, with few widely spaced radial striae, especially anteriorly; umbos low, wide; hinge plate extremely narrow, with one minute pseudocardinal in each valve; ligament internal, very short. Length up to 2.7 mm.

***Mantle*.** Large, reflected, covering ~ 95% of outer shell surface when fully extended, but not fully covering umbos; mantle can be almost completely retracted into the shell; reflected portion with low papillae; mantle near shell margin with longer tentacles; anterior end with large cowl, serrate on end; cowl can be greatly extended (Fig. 2E, F; Suppl. material 2).

***Foot*.** Of moderate size, ~ the length of the shell when fully extended, vermiform, with slight heel. The species is an active crawler, and can also attach to the host by byssal threads. The foot has been observed to frequently wrap around the urchin spines as the bivalve crawls.

***Ctenidia***. One demibranch on each side, comprised of ~ 30 narrowly spaced filaments in larger specimens.

***Brooding***. Up to ten shelled juvenile specimens observed brooding in the dorsal portion of ctenidia in mature specimens.

##### Distribution.

Only known from the type locality in False Bay, South Africa, and only found attached to the echinoid *Spatagobrissusmirabilis*; not observed free-living.

##### Commensal relationship and habitat.

Found crawling on the oral surface of the heart urchin *Spatagobrissusmirabilis*. This host species was found to be living in a specialized microhabitat of coarse gravel and half-buried cobbles or boulders (at least at the type locality associated with kelp forests). At the type locality in 2018, of 10 sampled heart urchins, all had associated *Brachiomya* on their oral surface. Densities of *Brachiomya* ranged from 38 to 172 specimens on a single host. Two other commensal species were also recorded on these same urchins, a small but very common unidentified amphipod of family Lysianassidae, and a large, scale worm (family Polynoidae), of which only a few specimens were found. The amphipod and polychaete species also both appear to be new to science.

##### Discovery.

Initially discovered via free-diving in 2016 at the type locality, collected by Jannes Landschoff and Craig Foster.

##### Etymology.

The name *ducentiunus* is from Latin, meaning “201.” The species was discovered while preparing and working on the ‘1001 Seaforest Species’ project, a research and storytelling program aimed at increasing awareness of regional kelp bed ecosystems colloquially referred to as ‘the Great African Seaforest’ (see www.seachangeproject.com). The number 201 was chosen as a unique identifier for the 1001 program, with the goal to link each hundredths species to a species described as new to science.

##### Comparisons.

The Pacific and Asian *Brachiomyastigmatica*, which is the only other known species in the genus, is more evenly rounded anteriorly, has a strong rust-colored stripe medially, lacks radial striae, and has more developed teeth.

#### ﻿ Montacuta

Taxon classificationAnimaliaGaleommatidaMontacutidae

Turton, 1822

C13519CA-467E-5A84-A7A7-C923089D199B

Montacuta Turton, 1822. Type species (subsequent designation) Ligula substriata Montagu, 1808. Recent, North Atlantic Ocean.

##### Description.

Shell small (length less than 5 mm), subovate to subelliptical, moderately thin, translucent to opaque, gaping ventrally in some; sculpture of commarginal striae and ribs, weak, widely spaced radial ribs in some; periostracum thin to thick, translucent to dark brown; hinge plate narrow, each valve with low anterior cardinal tooth; ligament internal, large, elongate; mantle sparsely papillate, reflected, covering some of outer shell surface; without mantle tentacles; foot elongate, thin, trigonal, heel absent; with one demibranch on each side.

##### Discussion.

While this genus is widely distributed in the North Atlantic, Mediterranean, and eastern Australia, this is only the second record from southern Africa. Kamenev (2008) provides a comprehensive description of the genus along with SEM images of the type species, *Montacutasubstriata*.

#### ﻿ Montacuta cf. substriata

Taxon classificationAnimaliaGaleommatidaMontacutidae

(Montagu, 1808)

A9F39F1A-1B0F-518F-88D0-13383DA6D997

Fig. 3A–F

Ligula substriata Montagu, 1808: 25.

##### Material examined.

Four specimens from 122 m off Agulhas Bank, ~ 110 km south off Mossel Bay (35.196°S, 22.056°E).

##### Description.

**Figure 3.**
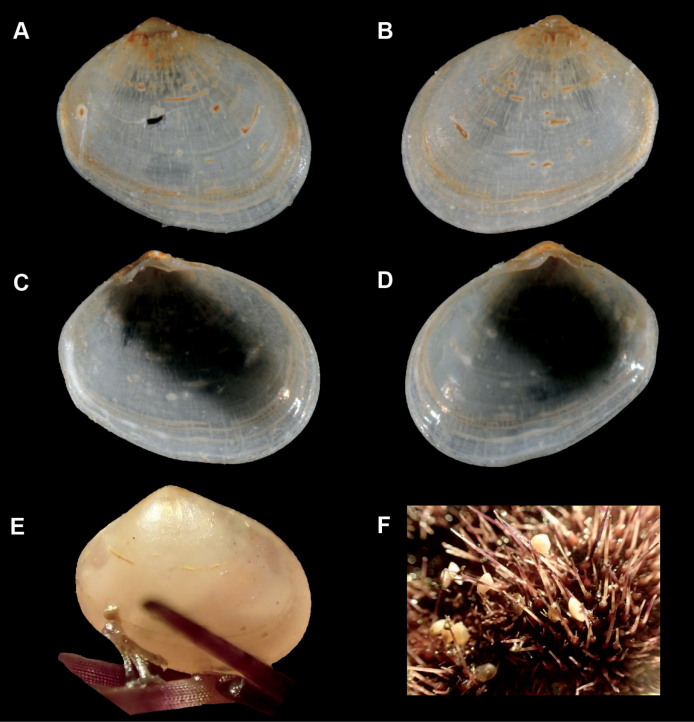
Montacutacf.substriata**A–D**SBMNH 467288, length = 3.0 mm, height = 2.2 mm **A** exterior of right valve **B** exterior of left valve **C** Interior of left valve **D** Interior of right valve **E** specimen attached to urchin spine **F** living animals attached to urchin *Spatanguscapensis*.

***Shell*** thin, fragile, moderately inflated, opaque; inequilateral, much longer anteriorly; anterior and posterior ends broadly rounded (Fig. 3A, B); shell margins only weakly gaping, if at all; prodissoconch length ~ 300 μm; dissoconch sculpture of commarginal striae, irregular widely-spaced radial striae, plus 1–3 low, broad, irregular radial undulations in some; umbos narrow, pointed, slightly projecting; hinge plate narrow; both valves with short, stout anterior cardinal tooth, and long, thin posterior cardinal tooth (Fig. 3C, D); ligament in oblique resilifer between cardinal teeth. Length up to 3 mm.

***Mantle*** not reflected.

***Foot***. Large, equal to or slightly longer than the length of the shell when fully extended, trigonal, without heel; long ventral byssal groove extending to end of smooth foot tip. Can attach to the host by byssal threads.

***Ctenidia***. With one demibranch on each side, comprised of ~ 20 widely-spaced filaments in larger specimens.

##### Type.

Lost; Devon coast, United Kingdom.

##### Commensal relationship and habitat.

Found crawling on the oral surface of the heart urchin *Spatanguscapensis* Döderlein, 1905. Up to 20 specimens have been observed byssally attached to the host.

##### Locality information.

Collected in 122 m off Agulhas Bank, ~ 110 km south off Mossel Bay (35.196°S, 22.056°E). Voucher specimens deposited as SBMNH 467288, SAMC-A096818, and UCM 60478.

##### Discussion.

*Montacutasubstriata* is a well-documented species in the North Atlantic (Oliver et al. 2016) and the Mediterranean (Gofas et al. 2011). Barnard (1964a) reported a single valve of this species in 100 fathoms (182 m). Cosel and Gofas (2019) did not report the species from tropical West Africa, nor have there been any other records from the African Atlantic or Indian Oceans. We acknowledge that there is likely limited gene flow between the populations of *Montacutasubstriata* in the North Atlantic and Cape Town, especially in light of the apparent absence of the species in tropical West Africa. However, the two populations match conchologically in all details although their hosts are different; *Spatanguscapensis* in South Africa and *Spatanguspurpureus* Müller, 1776 and *Enchinocardiumflavescens* Müller, 1776, in the northeast Atlantic. Unfortunately, we have been unable to extract DNA from our specimens from the Agulhas Bank, so we are unable to completely confirm this identification. It is possible that the South African specimens represent a new species.

#### ﻿ Kellia

Taxon classificationAnimaliaVeneroidaKelliidae

Turton, 1822

DA6A5CA8-B9A3-5859-8C9D-5D4D463D8F21

Kellia Turton, 1822. Type species (subsequent designation, Récluz 1844): Myasuborbicularis (Montagu, 1803). Recent, North Atlantic.
Chironia Deshayes, 1839. Type species (monotypy): Chironialaperousii Deshayes, 1839.
Diplodontina Stempell, 1899. Type species (monotypy): Diplodontinatumbesiana Stempel, 1899. Recent, Chile.

##### Description.

Shell subovate to ovate-elongate, inflated, subequilateral, equivalve; umbos prosogyrate; sculpture of commarginal ribs, striae, or growth checks; periostracum thin, translucent, green to yellow, dehiscent to adherent; hinge plate narrow; two small cardinal teeth in left valve, one cardinal tooth in right valve; one elongate, posterior lateral tooth in both valves; ligament internal, robust, in elongate resilifer.

##### Discussion.

There has been much taxonomic confusion with members of *Kellia*, especially in the southern hemisphere, and the genus needs a global revision. Kamenev (2004) documented many species of *Kellia* from the North Pacific and North Atlantic Oceans. The functional morphology of the genus has been documented by Oldfield (1961) and the sperm morphology by Jespersen and Lützen (2007)

#### ﻿ Kellia becki

Taxon classificationAnimaliaVeneroidaKelliidae

(W.H. Turton, 1932)

9A4973C1-529F-538F-B377-3EF5D37FD83B

Fig. 4A–H

Erycina becki W.H. Turton, 1932: 238.

##### Material examined.

Two specimens from at Glencairn, South Africa (34.162°S, 18.432°E).

##### Description.

***Shell*** ovate, thin, fragile, highly inflated, semi-translucent; subequilateral; umbos broad, moderately inflated; anterior and posterior ends broadly rounded; shell margins not gaping; periostracum thin, adherent, yellow, iridescent; external sculpture of fine commarginal striae; hinge plate narrow; right valve with one small cardinal tooth and one thin posterior lateral tooth, with large gap between them; left valve with two very small cardinal teeth and one posterior lateral tooth; ligament oblique, broad, in shallow resilifer. Length up to 6 mm.

**Figure 4.**
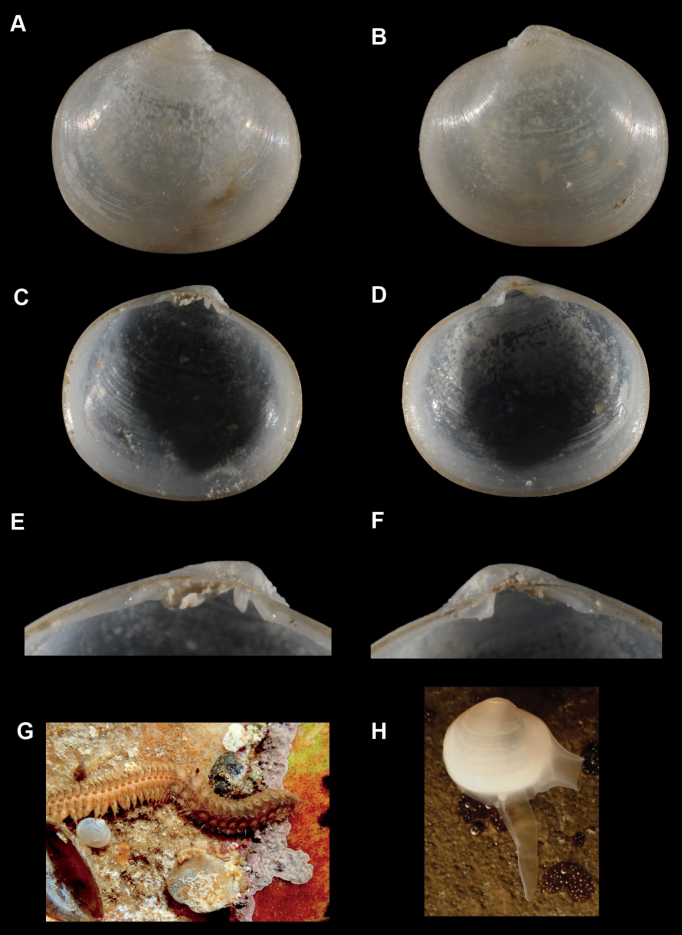
*Kelliabecki***A–F**SBMNH 669951, length = 5.6 mm, height = 4.9 mm **A** exterior of right valve **B** exterior of left valve **C** interior of left valve **D** interior of right valve **E** hinge of left valve **F** hinge of right valve **G** living animal on rock adjacent to polynoid polychaete, *Polynoescolopendrina***H** living animal with mantle and foot fully extended.

***Mantle*.** Translucent, reflected, extending well past shell margin dorsally, forming an extended facultative siphon posteriorly (Fig. 4H).

***Foot***. Long, thin, translucent, without heel (Fig. 4H).

***Ctenidia***. Specimens for internal examination not available.

##### Type.

ZC-M003209, Port Alfred, South Africa.

##### Commensal relationship and habitat.

Found on the undersides of rocks in intertidal pools. Although the errant polynoid polychaete, *Polynoescolopendrina* Savigny, 1822, is also visible in some of the images provided herein, we do not suspect any commensal relationship between the *Kelliabecki* and this polychaete and consider it to be a free-living, nestling species.

##### Comparisons.

Huber (2015) considered *Kelliabecki* to be the only member of this genus to be present in the Cape Town region. The Australian *Kelliarotunda* (Deshayes, 1856) had been recorded from South Africa by Bartsch (1915), W.H. Turton (1932), Barnard (1964a), and Kilburn and Rippey (1982), but these records were considered erroneous by Huber (2015), although he did mention the need to genetically compare these two species.

The specimens illustrated by Cosel and Gofas (2019: 486) in tropical West Africa as *Kelliasuborbicularis* (Montagu, 1803) are very similar conchologically to our specimens from the Cape Peninsula. Genetic studies on these two populations, as well as other Atlantic species of *Kellia*, are needed.

##### Locality information.

Collected intertidally from beneath boulders at Glencairn, on the east coast of the Cape Peninsula, South Africa (34.162°S, 18.432°E). Voucher specimens deposited as SBMNH 666951 and SAMC-A096819.

#### ﻿ Melliteryx

Taxon classificationAnimaliaGaleommatidaLasaeidae

Iredale, 1924

18F66592-2494-5873-9358-9427687190DC

Melliteryx Iredale, 1924. Type species (original designation): Erycinaacupuncta Hedley, 1902. Recent, Australia.

##### Description.

Shell subtrigonal, moderately inflated, subequilateral, equivalve; umbos narrow; sculpture of commarginal ribs, striae, or growth checks, with micro-pits in some; periostracum thick, tan, adherent, shiny to silky; hinge plate narrow; both valves with anterior and posterior lateral teeth; left valve with small central pseudocardinal directly below umbos, conjoined with anterior lateral tooth in some; right valve with small thickening near umbos; ligament internal, in elongate resilifer.

##### Discussion.

The type species of the genus, *Erycinaacupuncta* Hedley, 1902, was described from off New South Wales, Australia. Huber (2015) documents five additional species, including three from South Africa, one from the Indo-Pacific and one from New Zealand. See Discussion section below for additional comments on *Melliteryx*.

#### ﻿ Melliteryx mactroides

Taxon classificationAnimaliaGaleommatidaLasaeidae

(Hanley, 1857)

B35D34E6-3C9E-5850-866F-074FF73763C4

**Figure 5.**
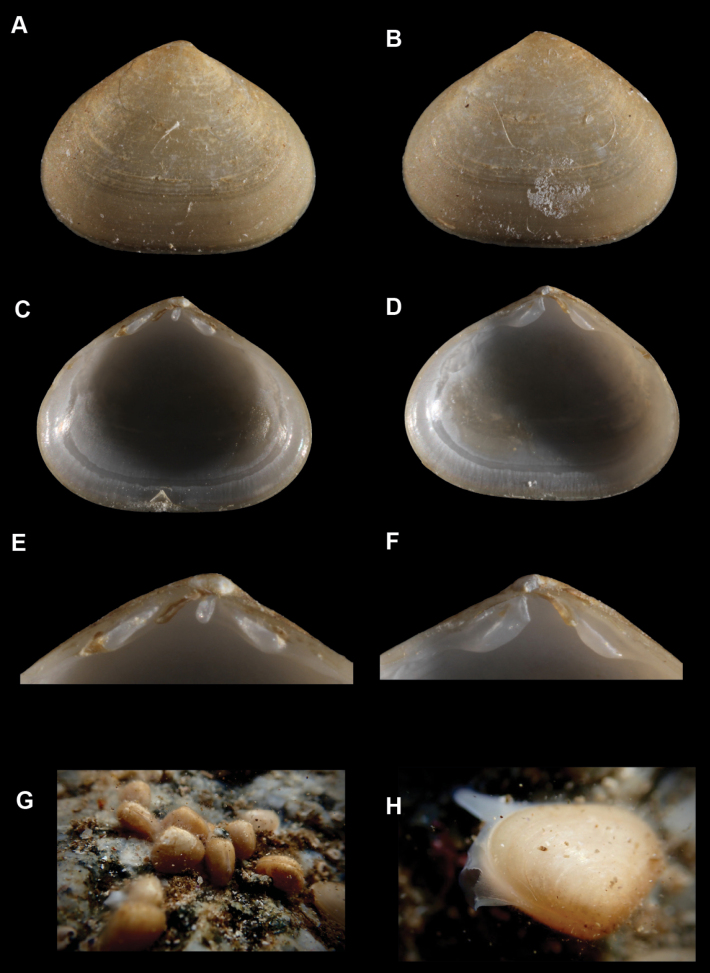
*Melliteryxmactroides***A–F**SBMNH 665156, length = 6.0 mm, height = 5.0 mm **A** exterior of right valve **B** exterior of left valve **C** interior of left valve **D** interior of right valve **E** hinge of left valve **F** hinge of right valve **G** living animals crawling on a rock **H** living animal with foot and mantle extended (note biofilm present in **G** and **H**).

Fig. 5A–H , Suppl. materials 3 , 4

Pythina mactroides Hanley, 1857: 340.

##### Material examined.

Three specimens from Miller's Point, False Bay, South Africa (34.231°S, 18.476°E).

##### Description.

***Shell*** trigonal, thick for size, moderately inflated, cream colored; subequilateral; umbos narrow, pointed; anterior and posterior ends broadly rounded; shell margins not gaping; periostracum thick, adherent, yellow to dark brown; exterior sculpture of fine commarginal striae, some with micro-pits; hinge plate broad; both valves with an anterior and posterior lateral tooth with a wide gap between them; left valve small central pseudocardinal tooth; anterior lateral tooth in right valve with small thickening near umbos; ligament oblique, narrow, in shallow resilifer. Length up to 6 mm.

***Mantle*.** Translucent, only slightly reflected, forming a facultative siphon posteriorly (see Suppl. material 3).

***Foot*.** Long, broad, translucent, with distinct heel. This species is an active crawler (see Suppl. material 3).

***Ctenidia*.** With one demibranch on each side, comprised of ~ 75 narrowly spaced filaments in larger specimens.

##### Type.

NHMUK 1967994, Cape of Good Hope, South Africa.

##### Commensal relationship and habitat.

Found in small groups of 10–20 animals, clinging to the underside of rocks in the lower intertidal. We have found no directly associated hosts.

##### Comparisons.

Huber (2015) documented two additional species of *Melliteryx* in South Africa, *Melliteryxjaeckeli* Huber, 2015, and *Melliteryxfortidentata* (Smith, 1904). *Melliteryxjaeckeli* has a much weaker hinge plate than *Melliteryxmactroides*, and we question whether this is the correct genus for the Smith species. *Melliteryxfortidentata* is inequilateral, with the umbos placed well off the center, compared to the subequilateral *Melliteryxmactroides*.

##### Locality information.

Specimens were collected in the intertidal at Miller's Point (34.231°S, 18.476°E). Voucher specimens were deposited as SBMNH 665156, SAMC-A096820, and UCM 60477.

##### Discussion.

We are following Huber (2015) and Cosel and Gofas (2019) with the placement of this species into the genus *Melliteryx*. As our molecular results indicate below there is little resolution of the genera due to lack of taxon sampling globally. With further genetic data it is possible that our South African species might fall into an undescribed genus.

Cosel and Gofas (2019) reported *Melliteryxmactroides* from tropical West Africa. The specimens they illustrate are more elongate than our specimens and have a weaker hinge plate. Additional study must be completed to determine if these are indeed the same species, or if the tropical West Africa specimens represent a new species.

On the shell exterior in living specimens of *Melliteryxmactroides* we observed a dense layer of filamentous biofilm (see Suppl. material 4). A similar biofilm was reported by (Gillan and De Ridder 1997) and (Gillan et al. 2000) in the North Atlantic galeommatid *Tellimyaferruginosa* (Montagu, 1808).

### ﻿Molecular results

**Figure 6.**
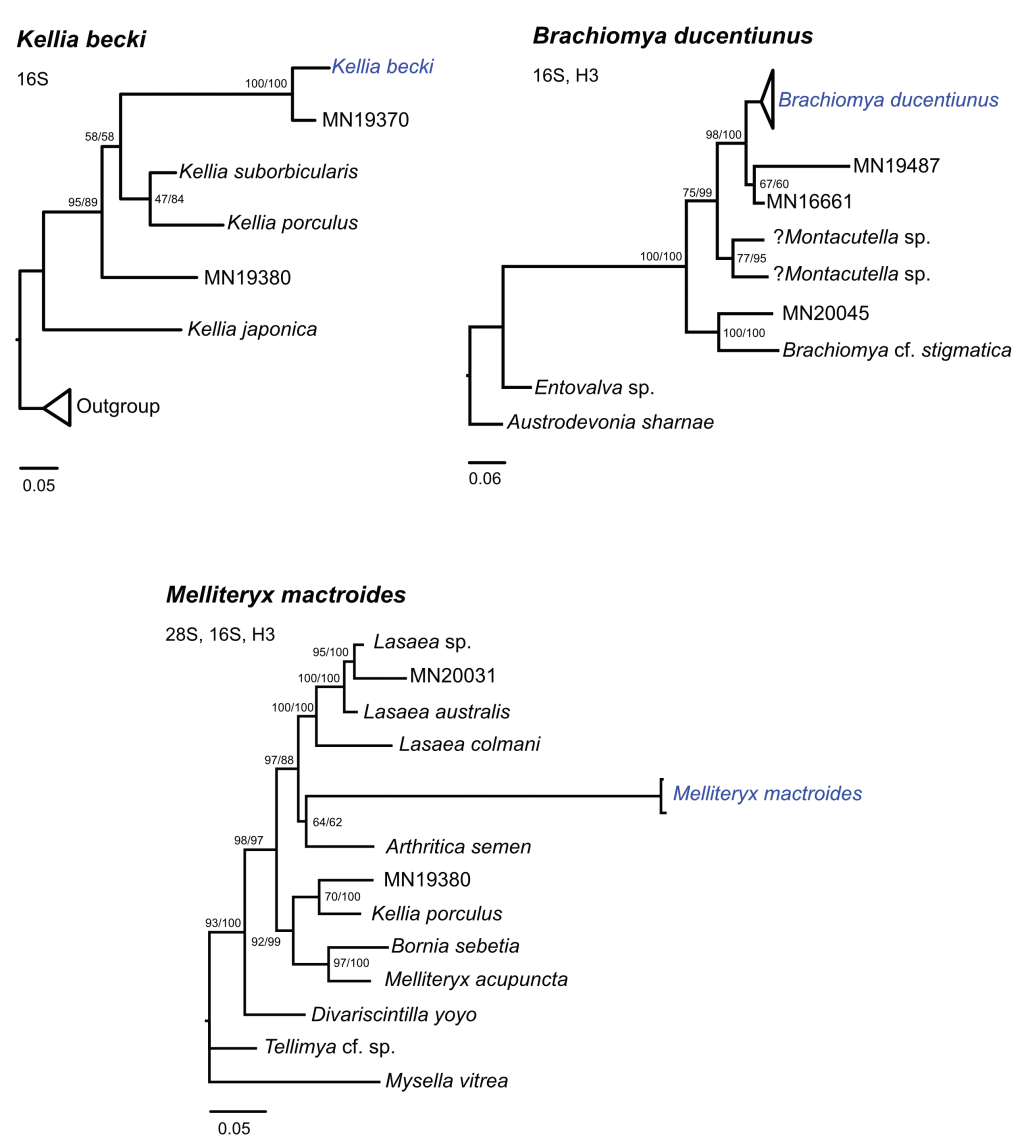
Phylogenetic positions of *Kelliabecki*, *Brachiomyaducentiunus*, and *Melliteryxmactroides*.

Phylogenetic positions of *Kelliabecki*, *Brachiomyaducentiunus*, and *Melliteryxmactroides* are shown in Fig. 6. Topologies from the Maximum Likelihood and Bayesian analyses were consistent for all species. *Kelliabecki* belong to a clade (Clade FS9 in Li et al. 2016) composed of other *Kellia* species, including *Kelliaporculus* Pilsbry, 1904, *Kelliajaponica* Pilsbry, 1895, and *Kelliasuborbicularis*. It was recovered as a sister lineage to an unidentified *Kellia* species collected from Madagascar.

*Brachiomyaducentiunus* belongs to a clade of sea urchin commensals (Clade CS2 in Li et al. 2016), including species from the genera *Montacutella* and *Brachiomya*. *Brachiomyaducentiunus* and two other unidentified sea urchin commensal species from Madagascar form a well-supported clade.

The phylogenetic position of *Melliteryxmactroides* was less resolved compared to the other two species, likely due to a lack of taxon sampling in this group. *Melliteryxmactroides* was recovered with high confidence as part of the FS1 clade in Li et al. (2016), which includes the genera *Lasaea* Brown, 1827 and *Arthritica* Finlay, 1926. However, its position within this clade is uncertain. *Melliteryxmactroides* is currently grouped with an Australian species *Arthriticasemen* (Menke, 1843) with low bootstrap or posterior support, and is quite divergent from *Arthriticasemen* based on the branch length. *Melliteryxmactroides* also does not appear to be closely related to *Melliteryxacupuncta* from Australia or ?*Tellimya* sp. from Japan.

The phylogenetic position of our *Montacutasubstriata* specimens could not be assessed due to unsuccessful PCR amplifications.

## ﻿Discussion

We have documented and described four species of galeommatid bivalves, including one new to science. We did not discover any of the galeommatids from eastern South Africa listed by Smith (1904) or Bartsch (1915). We did, however, locate a single species, *Kelliabecki*, which was described from Port Alfred by Turton (1932), and two species, *Melliteryxmactroides* and *Montacutasubstriata*, reported by Barnard (1964a).

The presence of the North Atlantic *Montacutasubstriata* in Cape Town represents an unusual disjunct distribution, as Cosel and Gofas (2019) did not report this or indeed any other members of the genus, from tropical West Africa. Future studies may prove the South African specimens to be a new species. The possible presence of European, northeast Atlantic species in South Africa was raised by Sowerby (1892) who reported eleven bivalve species as being identical to their British counterparts. Gradually these were dismissed although a few remain to this day such as *Talochlamysmultistriata* (Poli, 1795). A wider ranging molecular comparison of the north and south temperate faunas is warranted.

Our phylogenetic analyses provided us with intriguing, albeit sometimes confusing results (Fig. 6). *Kelliabecki* formed a clade with other *Kellia* members, but was found to be distinct. This confirms its current taxonomical placement within the genus *Kellia*.

*Brachiomyaducentiunus* belonged to a clade with *Brachiomyastigmatica* and also to an unidentified *Montacutella*Jespersen et al., 2004. It is also sister to an unidentified urchin-associated galeommatid from the biogeographically distant, Madagascar. Galeommatids are known for frequent evolutionary host switching and many groups do not exhibit high host fidelity (Goto et al. 2012; Li et al. 2016). The fact that echinoid-associated genera across a wide geographic range from a single clade indicates that this lineage exhibits exceptionally high host fidelity and perhaps host specialization. There is also a potential for bivalve-echinoid co-diversification to be detected in this group, which will require the host phylogeny to be constructed.

Our lack of sampling and understanding of the phylogeny of small commensal galeommatids is perhaps typified by the results with *Melliteryxmactroides* (Fig. 6). We compared two type species of galeommatids of the genera *Bornia* and *Melliteryx* to our South African samples. Interestingly, *Borniasebetia* (da Costa, 1830), the type species of the genus, has a sister relationship to *Melliteryxacupuncta*, which is the type species of *Melliteryx*. However, they are both quite distant from our South African species. Our South African bivalve is more closely aligned with the intertidal Australian *Arthriticasemen*. Ponder (2022) reviewed the Australian species of *Arthritica* and they have few similarities in shell morphology when compared to our species. Therefore, the taxonomical placement of our *Melliteryx* cannot be fully resolved until more taxon sampling is be done for this particular free-living clade. We are following Huber (2015) and Cosel and Gofas (2019) in the generic placement of this species until further genetic data is available.

Galeommatoidean bivalves and their hosts remain poorly known in South Africa and there are doubtless many more regional species that remain uncollected, especially those from deeper waters or commensal on other invertebrates.

## Supplementary Material

XML Treatment for Brachiomya

XML Treatment for Brachiomya ducentiunus

XML Treatment for Montacuta

XML Treatment for Montacuta cf. substriata

XML Treatment for Kellia

XML Treatment for Kellia becki

XML Treatment for Melliteryx

XML Treatment for Melliteryx mactroides

## ﻿Additional information

### Author ORCIDs

Paul Valentich-Scott https://orcid.org/0000-0003-0019-7643

Jannes Landschoff https://orcid.org/0000-0001-9836-1530

Ruiqi Li https://orcid.org/0000-0001-9428-6094

### Data availability

All of the data that support the findings of this study are available in the main text or Supplementary Information.

## Supplementary materials

Supplementary material 1 Three genetic markers from other galeommatoidean species belonging to closely related genera that were downloaded from GenBank and used for our analysis Data type xlsx https://binary.pensoft.net/file/1097716This dataset is made available under the Open Database License (http://opendatacommons.org/licenses/odbl/1.0/). The Open Database License (ODbL) is a license agreement intended to allow users to freely share, modify, and use this Dataset while maintaining this same freedom for others, provided that the original source and author(s) are credited.Paul Valentich-Scott, Charles Griffiths, Jannes Landschoff, Ruiqi Li, Jingchun Li

Supplementary material 2 *Brachiomyaducentiunus* sp. nov., crawling between urchin spines highlighting elongated and serrated cowl Data type mp4 https://binary.pensoft.net/file/1097717This dataset is made available under the Open Database License (http://opendatacommons.org/licenses/odbl/1.0/). The Open Database License (ODbL) is a license agreement intended to allow users to freely share, modify, and use this Dataset while maintaining this same freedom for others, provided that the original source and author(s) are credited.Paul Valentich-Scott, Charles Griffiths, Jannes Landschoff, Ruiqi Li, Jingchun Li

Supplementary material 3 *Melliteryxmactroides* in situ on rocks highlighting active crawling and large foot Data type mp4 https://binary.pensoft.net/file/1097718This dataset is made available under the Open Database License (http://opendatacommons.org/licenses/odbl/1.0/). The Open Database License (ODbL) is a license agreement intended to allow users to freely share, modify, and use this Dataset while maintaining this same freedom for others, provided that the original source and author(s) are credited.Paul Valentich-Scott, Charles Griffiths, Jannes Landschoff, Ruiqi Li, Jingchun Li

Supplementary material 4 *Melliteryxmactroides* in situ on rocks highlighting biofilm on outside of shell Data type mp4 https://binary.pensoft.net/file/1097719This dataset is made available under the Open Database License (http://opendatacommons.org/licenses/odbl/1.0/). The Open Database License (ODbL) is a license agreement intended to allow users to freely share, modify, and use this Dataset while maintaining this same freedom for others, provided that the original source and author(s) are credited.Paul Valentich-Scott, Charles Griffiths, Jannes Landschoff, Ruiqi Li, Jingchun Li
